# Mobile app and digital system for patients after myocardial infarction (afterAMI): study protocol for a randomized controlled trial

**DOI:** 10.1186/s13063-022-06463-x

**Published:** 2022-06-21

**Authors:** Bartosz Krzowski, Michał Peller, Maria Boszko, Paulina Hoffman, Natalia Żurawska, Karolina Jaruga, Kamila Skoczylas, Gabriela Osak, Łukasz Kołtowski, Marcin Grabowski, Grzegorz Opolski, Paweł Balsam

**Affiliations:** 1st Chair and Department of Cardiology, Medical University of Warsaw, 02-091 Warsaw, Poland

**Keywords:** Acute myocardial infarction, Telemedicine, Telehealth, Mobile application, Cardiac rehabilitation

## Abstract

**Background:**

Treatment of acute myocardial infarction has been the subject of studies over the past years. However, the initial months after myocardial infarction are crucial from the perspective of the patient’s prognosis. It is extremely important to take care of all cardiovascular risk factors and undergo a full rehabilitation program. Telemedical solutions are becoming more and more relevant in everyday practice. We describe a protocol of a study evaluating the use of the mobile application “afterAMI” in patients after myocardial infarction. The app offers an educational mode, calendar, vital signs diary, medication reminders, medical history card, and healthcare professional contact panel. It offers several solutions, which individually proved to be effective and improve a patient’s prognosis. Despite general promising results from previous studies regarding telemedical tools, there is a paucity of evidence when it comes to prospective randomized trials. Our aim was to perform a comprehensive evaluation of a newly developed mobile application in the clinical setting.

**Methods:**

A group of 100 patients with myocardial infarction on admission at the 1st Chair and Department of Cardiology, Medical University of Warsaw, will be recruited into the study. The project aims to assess the impact of the application-supported model of care in comparison with standard rehabilitation. At the end of the study, cardiovascular risk factors will be analyzed, along with rehospitalizations, the patients’ knowledge regarding cardiovascular risk factors, returning to work, and quality of life. In this prospective, open-label, randomized, single-center study, all 100 patients will be observed for 6 months after discharge from the hospital. Endpoints will be assessed during control visits 1 and 6 months after inclusion into the study.

**Discussion:**

This project is an example of a telemedical solution application embracing everyday clinical practices, conforming with multiple international cardiac societies’ guidelines. Cardiac rehabilitation process enhancements are required to improve patients’ prognosis. The evidence regarding the use of the mobile application in the described group of patients is limited and usually covers a small number of participants. The described study aims to discuss whether telemedicine use in this context is beneficial for the patients.

**Trial registration:**

ClinicalTrials.govNCT04793425. Registered on 11 March 2021.

**Supplementary Information:**

The online version contains supplementary material available at 10.1186/s13063-022-06463-x.

## Administrative information

Note: the numbers in curly brackets in this protocol refer to SPIRIT checklist item numbers. The order of the items has been modified to group similar items (see http://www.equator-network.org/reporting-guidelines/spirit-2727-statement-defining-standard-protocol-items-for-clinical-trials/).

**Table:** 

Title {1}	Mobile app and digital system for patients after myocardial infarction (afterAMI)
Trial registration {2a and 2b}.	ClinicalTrials.gov, NCT04793425, registered 11 March 2022.
Protocol version {3}	Version 4.0, 04.05.2022
Funding {4}	The work is carried out in the years 2020 to 2022, financed by the subsidy allocated to science, obtained by the Medical University of Warsaw
Author details {5a}	1) 1st Department of Cardiology, Medical University of Warsaw, Warsaw, Poland
Name and contact information for the trial sponsor {5b}	Not applicable; this trial does not have a sponsor.
Role of sponsor {5c}	Not applicable; this trial does not have a sponsor.

## Background and rationale {6a}

Cardiovascular diseases are the leading cause of death and a focal contributor to disability. Managing acute myocardial infarction (AMI) has improved significantly over the past years due to progress in both pharmacotherapy and invasive procedures. The mortality rate following AMI varies between countries, but an overall decrease has been observed [1]. Nevertheless, 12% of the patients die within one year after AMI [2]. Therefore, efforts should be made to optimize the cardiac rehabilitation process. It is crucial to focus on preventing future ischemic events by providing optimal care for patients at-risk [3]. Secondary prevention aims to control all cardiovascular disease (CVD) risk factors, which may be challenging in everyday practice. Jankowski et al. reported that only 2.9% of patients with coronary artery disease (CAD) have all CVD risk factors adequately controlled corresponding to values recommended in the guidelines [4]. Proper CVD risk factor control remains a challenge in the real-world setting.

Several efforts are being made to improve patients’ prognosis. The latest approach to improve cardiac rehabilitation is the use of novel telehealth-based solutions. Over 3.2 billion smartphones are used globally, and the mobile applications market is expected to grow by 18.4% between 2018 and 2026. Therefore, enhancing cardiac rehabilitation by mobile application support may be a promising tool. Telemedicine has proved to be an effective solution in clinical scenarios. Widmer et al. demonstrated that augmentation of usual cardiac rehabilitation with an online and smartphone-based program improved CVD risk factor management and reduced rehospitalizations or emergency department visits by 40% (*p*<0.05) [5]. Naturally, not all cardiac patients are capable of using smartphones. Gallagher et al. reported that 54.6% of cardiac patients eligible for attending cardiac rehabilitation used technology for health purposes. Patients used it to access information on health conditions and medications mainly [6]. Coorey et al. concluded in the meta-analysis that mobile applications have a beneficial influence on CVD risk factors control, but more scientific evidence is required to enhance the implementation of telemedicine into clinical practice [7].

Although several international cardiac societies recommend telemedicine use [8, 9], evidence-based conclusions are required to adjust specific telemedical tools individually to the patient and improve the prognosis. The influence of mobile application support on cardiac rehabilitation in a European setting is yet to be studied.

### Objectives {7}

This study will aim to determine the effect of mobile application-supported cardiac rehabilitation on CVD risk factors control, rehospitalization, emergency department visits, quality of life, and the ability to return to work. We hypothesized that cardiac rehabilitation enhancement with the mobile application would improve the prognosis expressed by CVD risk factors management and the patient’s quality of life.

## Methods

### Study setting {9}, Eligibity criteria {10}, Who will take informed consent? {26a}, Additional consent provisions for collection and use of participant data and biological specimens {26b}, Explanation for the choice of comparators {6b}, Trial design {8}, Provisions for post-trial care {30}

This protocol is a randomized, open-label, superiority, interventional study with two arms. Participants will be randomized to (1) a control group (CG) with standard cardiological care or (2) a mobile application-supported interventional group (IG). The participants will continue with traditional care after the trial is finished.

This single-center study will be carried out at the 1st Department of Cardiology at the Medical University of Warsaw, an academic, public hospital in the capital of Poland. Cardiologists and fellows of cardiology will conduct all study-related procedures. The Department ensures all treatment options for patients with AMI and during their cardiac rehabilitation process. It is regarded as the leading Department of Cardiology in Poland. The anticipated number of eligible participants is 100.

Inclusion criteria:

- Signing the informed consent to participate in the study
- Hospitalization due to myocardial infarction, based on the Guidelines on Fourth Universal Definition of Myocardial Infarction [10]
- Owning a mobile device with Internet access and the Android/iOS operating system
- Age ≥18 years old
- Positive results of a test verifying the basic skills of using mobile applications (Supplementary material 1)

Exclusion criteria:

- Life expectancy shorter than 6 months due to non-cardiac illness (Those with malignant tumors, severe mental illness, and/or uncontrolled systemic diseases were excluded from the present study)
- Negative test results, regarding everyday mobile application use
- Lack of signed informed consent
- Age <18 years old
- Pregnancy or breastfeeding
- Lack of a mobile device with Internet access and the Android/iOS operating system

Participant recruitment will occur daily from Monday to Friday. A study team member will approach every patient presenting with AMI, and the inclusion criteria will be assessed. The study design will be thoroughly described to the patient, including all potential benefits, harms, and ethical implications. Each eligible patient will be proposed to enter the study. Patients will be given time to ask questions and all doubts will be clarified before inclusion into the study. If the patient agrees to participate in the research, the informed consent will be signed in 2 copies: one for the participant and one for the research archives. The informed consent will explain how participant data and blood samples will be handled and where they will be sent. This trial does not involve collecting biological specimens for storage. Every participant will receive a note with a summary of the study design.

Every year approximately 400 patients are hospitalized due to acute myocardial infarction. However, a significant part of those patients are unable to use a mobile application and therefore they do not meet one of the main inclusion criteria. Going further, only some of the patients who meet inclusion criteria are willing to take part in a trial. The recruitment began in December 2020. Approximately 7 patients per month are included in the trial. Last patient recruitment is expected in Q1 2022.

## Assignment of interventions: allocation, blinding

### Sequence generation {16a}, Concealment mechanism {16b}, Implementation {16c}, Who will be blinded {17a}, Procedure for unblinding if needed {17b}

**Fig. 1:**
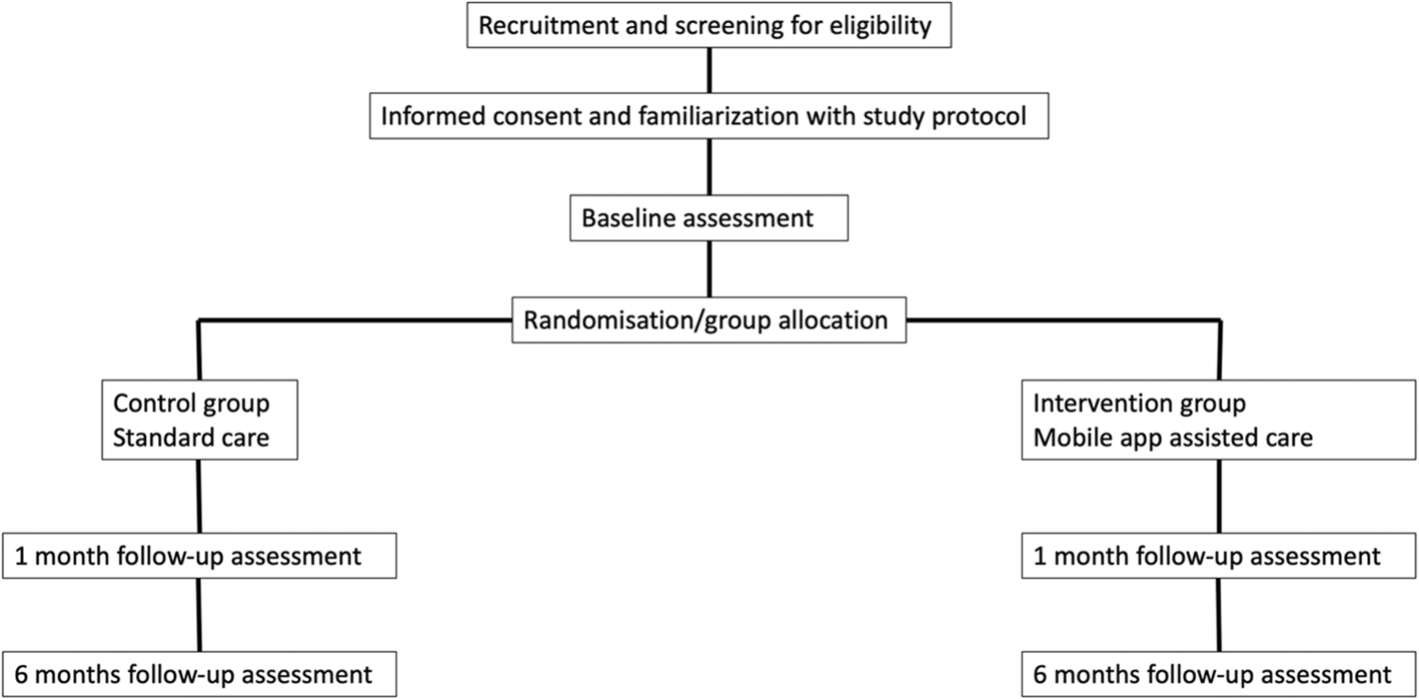
Study flow chart

This is an open-label study. Randomization will be performed with an online tool by an independent statistician available at (www.randomizer.org). A hundred sets will be generated, each with a number (1 for CG and 2 for IG). All allocations to CG and IG will be executed before the study begins. The list of subsequent allocations will not be visible for the recruiting physician until the initial eligibility assessment of the patient and obtaining the patient’s consent for study participation. After collecting the initial documentation, the physician will be unblinded and will receive the group allocation information from the principal investigator. It should be underlined that the person who checks for inclusion criteria and introduces the patient into the trial protocol is blinded until patient agreement. Figure 1 shows the study design flow chart, describing all the steps of the study (Fig. 1). The investigator performing statistical analysis will be blinded, as well as nurses collecting blood samples on the follow-up visits. Taking into consideration the use of the mobile application in everyday practice, it is impossible to blind physicians performing follow-up visits. It could be regarded as ethically doubtful, because based on the data provided via mobile application, clinical decisions can be made (i.e., blood pressure treatment augmentation).

### Intervention description {11a}, Criteria for discontinuing or modifying allocated interventions {11b}, Strategies to improve adherence to interventions {11c}, Relevant concomitant care permitted or prohibited during the trial {11d}

Patients in the intervention group will be granted access to the mobile application (afterAMI) during the rehabilitation process. Access to the app is what differentiates the groups. afterAMI app offers several features described below. What is more, a dedicated web page will be used by medical professionals to improve everyday clinical work organization and enable better contact with the patients.

Patients in the intervention group will also be given access to educational data about their diseases. Every educational chapter was prepared by a cardiologist experienced in managing patients after MI.

Additionally, every patient will regularly receive messages with notifications about the recommended lifestyle interventions and promoting adherence to the therapy. The notes focus mainly on cardiovascular risk factors and ways of controlling them. We made na effort to emphasize the importance of everyday lifestyle changes in terms of minimizing the risk of potential ischemic events in the future. The role of physical activity and drug adherence was frequently emphasized. All notes were prepared based on recommendations provided by the European Society of Cardiology [11]. An optimal scheme for message quantity and structure has not been described yet in the case of cardiac patients. In a recent meta-analysis, Bashi et al. concluded that the results of mobile application-assisted patient education generally show a positive, promising result. However, due to poor reporting quality and considerable heterogeneity of applied interventions, further studies are required in order to develop a comprehensive, optimal educational scheme [12]. In another paper, a cardiac telerehabilitation program augmented by a short message service (SMS) delivered to patients once a week, resulted in both physical fitness and quality of life improvements [13]. Based on those reports, the authors of this trial decided to send 2 messages weekly. The notifications are also considered as a strategy to improve adherence to the recommendations.

Another essential feature of the application is a panel dedicated to reporting patients’ vital signs (blood pressure, heart rate, weight, saturation, and glycemia), which will be daily analyzed, and if necessary, a short message will be sent to the patient, advising to present to the primary healthcare clinic or emergency department. Patients will be instructed to report their parameters daily, in case of manually entered data. If the patient has a wrist-worn wearable device, compatible with ‘health; application on iOS or Android, then this data will also be presented in the afterAMI app. For instance, in the case of patients with smartwatches allowing for heart rate measurement with photopletyspography or ECG, these parameters (heart rate, blood pressure, body weight, saturation, and physical activity) will automatically be transferred into the app, directly after measurement. Patients with a pressure gauge without a connection to the mobile phone will have to enter the data manually. Naturally, there is room for error associated with adding the value of a particular parameter, but it can also happen during traditional “notebook” notes. A possible clinical scenario is the detection of an alarming rapid increase in bodyweight, which might foreshadow incoming heart failure exacerbation. Another potential use of the application is reporting rapid pulse. Then, new-onset atrial fibrillation might be suspected. In every case requiring medical confirmation, the patient will be referred to the nearest emergency room. However, all patients will be informed, that they should immediately present to the nearest emergency unit or contact the emergency services, in case of recurring angina or any other acute complaints.

Additionally, the application will send notifications with reminders to take drugs. This solution has been previously evaluated in many studies and proved to be a successful tool in increasing adherence to therapy [14].

Moreover, the application includes a module with air pollution parameters measured amidst the localization set up. If they exceed the alarming levels, the patients will be notified, and it will be suggested to minimize outdoor physical activities

Additionally, a medical history card will be created for each patient, based on the discharge documents from the hospital. This solution aims for the patient always to have brief information about undergoing coronary interventions. This knowledge might be crucial for the physicians performing subsequent percutaneous coronary interventions (PCI) in the future and could potentially decrease time-to-balloon.

Finally, the application offers a contact panel to text message and call the cardiologists at the hospital. We believe that this will translate into better work organization, better time management (as fewer consultations are likely to be missed by patients), and increased patient safety. All patients randomized to IG will be thoroughly trained in application features and capabilities before discharge.

On the contrary, patients randomized to the CG will be provided with the best available care, based on current guidelines and standards of care [15]. What is more, all patients will be provided with intense medical care supervision, as every patient included in the study will have two additional cardiological consultations.

Rehabilitation programs are recommended for every patient hospitalized due to MI. It has been underlined, that it improves a patient’s prognosis. However, the final decision on the participation is up to the patient. It should be noted, that the study is carried out during the COVID-19 pandemic. Some patients are refusing to participate in the rehabilitation program in fear of infection. However, owing to the randomization, the number of patients who refused to attend rehabilitation is expected to be similar between the groups. Rehabilitation programs include regular cardiac consultations with an experienced physician, psychological sessions, exercise training, stress management programs, and dietary recommendations. The patient is offered versatile support for one year after MI. Patients who refuse to take part in the rehabilitation program are managed by a general practitioner. Owing to the randomization, patients’ distribution is expected to be similar in both groups.

All data implemented into the application was prepared by experienced cardiologists with considerable experience in both, clinical practice as well as eHealth use.

The market currently offers a wide variety of mobile applications offering simple features to the general population (i.e., blood pressure diaries). However, as far as the authors know, this is one of the very first digital solutions to combine several previously tested features. Moreover, the number of mobile applications dedicated to MI patients is limited. What is more, the first few weeks and months after discharge can be a considerable challenge in everyday activities, therefore it is crucial to recognize the need to support the patient in this period. The described digital tool should be regarded as an opportunity to improve patients’ prognosis, augmenting the traditional approach with standard practice. The only criterion for discontinuing is the participants’ request. What is more, concomitant care is neither permitted nor prohibited in the trial. At the end of the study, the participants will receive a full report with the results of their assessments after the data is analyzed. At the end of the study, the principal investigator will contact the participants to provide them with final educational materials and information regarding secondary cardiovascular prevention.

### Outcomes {12}, Participant timeline {13}, Plans to promote participant retention and complete follow-up {18b}

All 100 patients will be observed for 6 months after discharge. The endpoints will be assessed twice, during two control visits, in 1- and 6-months, after enrollment. Patients will be called to schedule the control visit’s date after discharge. Additionally, patients in the IG will receive a notification in their mobile app reminding them about the upcoming ambulatory visits.

**Fig. 2:**
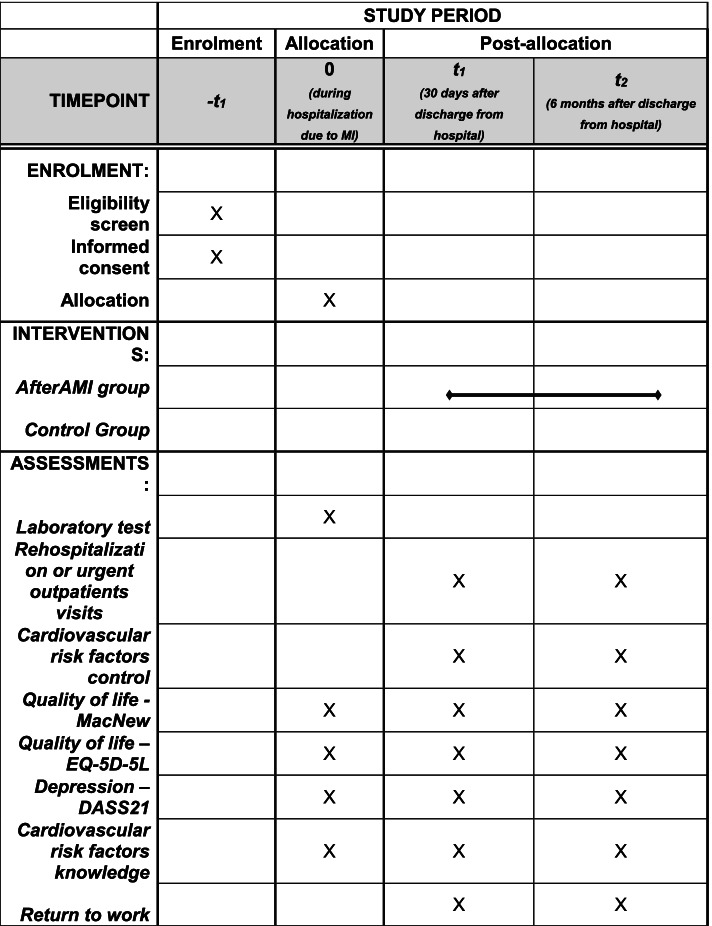
Recommended SPIRIT figure with participant timeline

Figure 2 shows the recommended SPIRIT figure with the participant timeline. Primary outcome includes both need for rehospitalization and/or urgent outpatient visit and assessed between baseline and 6-month control visit. There are five secondary outcomes related to cardiovascular risk factors control: blood pressure, body mass, nicotinism, dyslipidemia and need for rehospitalization and/or urgent outpatient visit assessed between baseline and 1-month control visit. Detailed target values regarding risk factors control have been presented in Table 1 based on ESC Chronic Coronary Syndrome guidelines [16]. Each value will be categorized as met or not. Secondary outcomes will also include quality of life and depression severity assessment (MacNew, EQ-5D-5L, and DASS-21 questionnaires), cardiovascular risk factors knowledge (CVD risk factors identification recommended BP values, desired lifestyle intervention identification). and return to work in case of professionally active patients.

**Table 1:** Risk factors targets for endpoint assessment

Risk factor		Target level
LDL-c level	No history of MI within 2 years prior	LDL-C lowered by at least 50% from baseline and to <1.4 mmol/L (<55 mg/dL)
	History of MI within 2 years prior	LDL-C of <1.0 mmol/L (<40 mg/dL) in patients who have experienced a second vascular event within 2 years
Body mass index		18.5–24.9 (kg/m^2^)
Systolic blood pressure	General	120–130 mmHg
	Older (aged >65 years)	130–140 mmHg
Nicotinism		Currently non-smoker

*Abbreviations*: *MI* myocardial infarction, *LDL* low-density lipoprotein cholesterol

Further data collection will cover demographic parameters (like sex, age), as well as laboratory test results, and types of prescribed pharmacotherapy.

#### Secondary outcome measures

##### Cardiovascular risk factors

Blood pressure

All patients at discharge are asked to measure and note blood pressure values daily. The mobile application-derived mean blood pressure values covering 5 days prior to their visits will be averaged for the IG patients. The mean blood pressure values of CG-comprised patients will also be averaged, but based only on the presented written notes. Additionally, meeting guidelines-based recommended values will be checked in both groups. Hypertension is one of the main cardiovascular risk factors, with a significant prevalence of 1.13 billion worldwide [17]. Hypertension control in patients after MI is crucial and correlates with patients’ prognosis [18].

Body mass

All patients will be weighed on admission tl and during control visits 1 and 6 months after discharge. Weight change will be measured. Maintaining a healthy body weight is one of the fundamental aspects of preventing cardiovascular diseases and an essential treatment element after an MI.

Nicotinism

All patients will be asked about smoking on admission and during control visits 1- and 6-months after discharge. Smoking cessation is one of the main goals of patients after MI. Quitting smoking is necessary to reduce the risk of another ischemic incident. It has been documented that smoking cessation results in 50% reduction in the risk of experiencing another MI [19].

Dyslipidemia

All patients after MI will have their cholesterol levels measured during hospitalization. Subsequent cholesterol level measurements will be performed during control visits. According to ESC guidelines, different groups of patients have different LDL cholesterol target values, which should be met during the rehabilitation process [20]. Lowering LDL cholesterol levels correlates with a better prognosis after MI [21].

Quality of life

Quality of life will be assessed with four questionnaires. The MacNew questionnaire contains 27 questions [22]. The scoring of the MacNew is as follows, the maximum score in every domain is 7 [high quality], and the minimum is 1 [poor quality]. The quality of life is assessed in the context of physical, emotional, and social aspects. The the EQ-5D-5L questionnaire refers to 5 aspects: mobility, self-care, usual activity, pain/discomfort, and anxiety/depression [23]. Every segment is assessed based on a 5 level scale-LEVEL 1: indicating no problem; LEVEL 2: indicating slight problems; LEVEL 3: indicating moderate problems; LEVEL 4: indicating severe problems; LEVEL 5: indicating an inability to/extreme problems. DASS 21 scale will be used to assess depression, anxiety, and stress [24]. The DASS is a quantitative measure of distress along the 3 axes of depression, anxiety, and stress. Seven questions are assigned to every aspect: depression, anxiety, and stress. Each question has 4 possible answers:

0- Did not apply to me at all - Never
1- Applied to me to some degree, or some of the time - Sometimes
2- Applied to me to a considerable degree, or a good part of the time - Often
3- Applied to me very much, or most of the time - Almost always

Seven questions are assigned to every aspect: depression, anxiety, and stress.

Higher result in each section contributes to higher severity in depression, anxiety, and stress.

Cardiovascular risk factors knowledge

Cardiovascular risk factors knowledge will be assessed with a previously prepared questionnaire (Supplementary material 2).

Return to work

In the case of previously working patients, the likelihood of returning to work will be assessed, as well as the timing of returning to work will be counseled.

### Sample size {14}

Currently, data regarding the achieved reduction of rehospitalizations or prelevance of urgent visits associated with mobile application use remains limited. Most previous studies were conducted on smaller populations. The sample calculation was based on Widmer and colleagues’ [5] study, considering rehospitalization and urgent ambulatory visits—comparing the effects of an online and smartphone-based program with standard rehabilitation on the mentioned endpoint. A 40% decrease in the primary endpoint was observed. Fifty percent of patients in the control group and 20% in the interventional arm were rehospitalized or visited the emergency department.

An online calculator (https://clincalc.com/) was used to determine the sample size, assuming the power of 80% and significance of 5%. A total of 76 patients (38 per group) were required. However, considering a possible lost-to-follow-up group and possible dropouts, a decision to recruit 100 patients was made. The reason for the dropout is lack of consent or inability to attend control visits or consent withdrawal.

## Data collection and management and confidentiality

### Plans for assessment and collection of outcomes {18a}, Data management {19}, Confidentiality {27}, Plans for collection, laboratory evaluation and storage of biological specimens for genetic or molecular analysis in this trial/future use {33}

The assessment of outcomes will be carried out at the 1st Department of Cardiology, Medical University of Warsaw, while the rehabilitation will be conducted according to a scheduled program. An experienced cardiologist familiar with the study protocol will conduct all the control visits, which will take place in the cardiac ambulatory clinic. Blood samples will be sent to the local laboratory. All blood samples will be tested in the laboratory according to the locally implemented standards and subsequently utilized. There are no plans for future blood use. No other biological specimen will be tested during this trial. Patients will be asked to fulfill the MacNew, EQ-5D-5L, and DASS21 questionnaires. The highest data quality is one of the authors’ primary goals while conducting a trial. Every information will be entered into a database and subsequently checked by another investigator. Every investigator will have a valid good clinical practice certificate.

All data collected during the study and medical documents will be protected and stored in a room dedicated to clinical trial records. All electronic materials will be duly stored in the principal researcher’s computer protected with a password known only to the principal researcher. Additionally, a backup in the cloud will be performed after new data collection.

The highest level of confidentiality will be applied. The participants’ data will be kept separately from any identifying information. According to good clinical practice, all investigators will make every effort to keep the sensitive data confidential.

## Statistical methods

### Statistical methods for primary and secondary outcomes {20a}, Methods for additional analyses (e.g. subgroup analyses) {20b}, Methods in analysis to handle protocol non-adherence and any statistical methods to handle missing data {20c}, Interim analyses {21b}

In terms of the endpoints, we will look at the frequency of the events 6 months after the discharge, since we believe that due to randomization the groups will be similar at the beginning. No other (including no interim) analyses will be performed in the present study. Regarding secondary endpoints, the change from baseline will be assessed and the frequency of the events in case of rehospitalizations and/or urgent ambulatory visits after 1 month. The distribution of continuous variables will be estimated using the Shapiro-Wilk test. Continuous variables presented with normal distribution will be presented as mean values and standard deviations (SD). All continuous variables presented non-normal distribution will be demonstrated as median values and interquartile ranges. In the case of variables with a normal and non-normal distribution, the groups will be compared using the Student's t-test and the non-parametric Mann-Whitney U test. The comparison of qualitative variables between the groups will be performed using the Fisher exact test. In order to compare changes in the values of continuous variables over time, the analysis of variance will be performed. To compare the outcome of the patients, the Kaplan-Meier estimators will be utilized. For quantitative variables, the change from baseline will be assessed. Additionally, in order to diminish differences in sex and age related to group size, propensity score matching analysis will be performed as well. We also plan to conduct a subanalysis in the group of patients who attended rehabilitation, as well as in those who did not attend.

A per-protocol analysis will be performed after completing all of the follow-up visits. In the analysis, we will include all patients who meet inclusion criteria and sign informed consent regardless of the follow-up completion. Statistical calculations will be performed twice; after obtaining data from the first follow-up visit from all patients and after the final follow-up, 6 months after discharge. In case of missing data, patients will be excluded from the particular analysis.

### Plans to give access to the full protocol, participant-level data and statistical code {31c}

Access to the data sets and statistical code is not planned for this study. However, this material might be available upon an adequately justified request to the corresponding author while maintaining participants’ anonymity.

## Oversight and monitoring

### Composition of the coordinating center and trial steering committee {5d}, Composition of the data monitoring committee, its role and reporting structure {21a}, Adverse event reporting and harms {22}, Frequency and plans for auditing trial conduct {23}, Plans for communicating important protocol amendments to relevant parties (e.g. trial participants, ethical committees) {25}

The research center is coordinated and managed by GO and the researcher MG. The principal investigator-BK will direct and continuously monitor trial conduct.

No additional external monitoring committee is considered for this study. The principal investigator will meet monthly with all the researchers involved in this study via an online platform (Zoom) to discuss the research progress and solve possible issues. Researchers are instructed to immediately report any issues to the principal investigator, who will subsequently organize an additional committee meeting and inform the board review committee from the Medical University of Warsaw and the Ethics Committee of the Medical University of Warsaw, Warsaw, Poland, when appropriate. Additional auditing will be conducted on request from the Ethics Committee of the Medical University of Warsaw.

As serious adverse events of mobile application usage have not been described so far, we do not expect the need for adverse event reporting However, any adverse events will be reported and thoroughly documented and presented in the study summary. Furthermore, monthly reports regarding any potential adverse events and protocol violations will be prepared

Any protocol amendments will be reported to and approved by the Ethics Committee of the Medical University of Warsaw. All modifications will be updated at clinicaltrials.gov by the principal investigator (BK). Any important protocol modifications will be communicated to the investigators and patients verbally.

### Dissemination plans {31a}

Study outcomes will be reported at both local and international cardiological conferences. The final study results will be submitted to a peer-reviewed indexed scientific journal within the 3 years after the last patient’s enrollment.

## Discussion

This study aims to investigate the effects of mobile application-assisted cardiac rehabilitation after MI on rehospitalization rate, cardiovascular risk factors control, and patients’ quality of life. Considering the alarmingly high 12-month mortality rate after MI, there is a considerable need for improving the rehabilitation process by intensifying and optimizing cardiovascular risk factors’ control. Standard approaches consisting of pharmacotherapy and recommended lifestyle modification have been thoroughly studied and included in the current guidelines [3]. Novel methods supporting the patient’s involvement and adherence are of the highest importance.

Telemedicine is a rapidly growing branch of heatlhcare, with numerous novel technologies proposed for improving the diagnostic, therapeutic, and rehabilitation process. Cardiology is one of the primary beneficiaries of the newly implemented tools. Several cardiac guidelines recommend enhancing everyday clinical practice with mHealth solutions [9, 25, 26]. Even though there is a varirty of mobile applications dedicated to patients, only a few were validated in clinical settings, with regard to the mentioned endpoints. Additionally, many of them were not developed by clinicians. It is crucial to establish whether such an approach, based on digitally supported rehabilitation, may translate into a better prognosis through facilitating more adequate cardiovascular risk factors control. In previous studies, the use of mobile applications has been associated with a reduction of the rehospitalization rate after MI, but the data were collected only in a smaller sample of patients and in different healthcare systems [5]. Johnston et al. reported that using a simple, mobile application results in better self-reported drug adherence and may correlate with lifestyle changes and quality of life [27]. However, the discussion regarding the use of mobile applications in cardiac patients is still ongoing.

Despite several strengths, certain limitations of this study should be considered. Firstly, we will be unable to assess the mortality rate due to a short observation period and small sample size (both due to organizational issues). However, we do not expect this parameter to differ between groups. Additionally, this single-center analysis might be biased due to internal protocols, which might differ in other clinics.

Nonetheless, our project stands as a practical example of implementing modern solutions to improve patients’ prognoses. If our assumptions regarding the potential beneficial effects of using the afterAMI application appear trustworthy, this study will provide a stronger voice in discussing broader telemedicine usage in everyday clinical practice.

## Trial status

Recruiting.

Version 3. February 21st, 2022.

Date recruitment began: December 1, 2020. Approximate date when recruitment will be completed: March 31, 2022.

## Supplementary Information

**Additional file 1: Supplementary material 1.** Test verifying the basic skills of using mobile applications.

**Additional file 2: Supplementary material 2.** Cardiovascular risk factors’ knowledge test.

## Abbreviations

AMI: Acute myocardial infarction
CVD: Cardiovascular disease
CAD: Coronary artery disease
CG: Control croup
IG: Interventional group
PCI: Percutaneous coronary intervention
