# Supplementary material for: Mobile app and digital system for patients after myocardial infarction (afterAMI): study protocol for a randomized controlled trial
Source: Trials. 2022 Jun 21;23:522. doi: 10.1186/s13063-022-06463-x (PMC9210045; doi:10.1186/s13063-022-06463-x)
Supplement: Supplementary file 1 — Additional file 1: Supplementary material 1. Test verifying the basic skills of using mobile applications. [file 13063_2022_6463_MOESM1_ESM.docx]

Supplementary material 1. Test verifying the basic skills of using mobile applications

Please mark the apps you use:

□ GPS/ Sat nav (e.g. Google maps)

□ Finance/mobile banking apps

□ Social media apps (e.g. Facebook, Messenger, Instagram)

□ Transport-related apps (e.g. Bolt, Uber)

□ Weather forecast apps

□ Shopping apps (e.g. Allegro, Zalando, Olx, Vinted)

□ Food delivery apps (e.g. Pyszne.pl, Uber Eats, Glovo)

□ Video apps (e.g. YouTube, Netflix)

□ Music/ podcast apps (e.g.. Spotify, Tidal, Soundcloud)

□ Photo editing apps

□ Health/ life-style related apps (e.g. calorie counters)

□ Grocery shopping apps (e.g. Frisco, Lidl Plus, Żappka, Twoja Biedronka)

□ Apps with cooking recipes

□ Sports apps (e.g. Endomodo)

Results:

At least 2 marked points qualified patient as able to use mobile applications.
