# Supplementary material for: Mobile app and digital system for patients after myocardial infarction (afterAMI): study protocol for a randomized controlled trial
Source: Trials. 2022 Jun 21;23:522. doi: 10.1186/s13063-022-06463-x (PMC9210045; doi:10.1186/s13063-022-06463-x)
Supplement: Supplementary file 2 — Additional file 2: Supplementary material 2. Cardiovascular risk factors’ knowledge test. [file 13063_2022_6463_MOESM2_ESM.docx]

Supplementary material 2. Cardiovascular risk factors' knowledge test

1. Are you familiar with the term „cardiovascular risk factor”?
   A. Yes
   B. No
2. Please name all cardiovascular risk factors that you know of:
   …………………………………………………………………………………………………………………………….
   …………………………………………………………………………………………………………………………….
   …………………………………………………………………………………………………………………………….
3. Fill in the gaps. Hypertension is diagnosed when in at least two measurements (on at least two separate visits) the patient’s mean arterial blood pressure is equal or higher than …. for systolic blood pressure and/or …. for diastolic blood pressure The correct answer:
   A. 120; 80 mm Hg
   B. 180; 90 mm Hg
   C. 140; 90 mm Hg
   D. 150; 75 mm Hg
4. Diabetes can lead to myocardial infarction. Which symptoms indicate high blood glucose levels?
   A. increased thirst
   B. frequent urination
   C. impaired vision
   D. all answers are correct
5. What is the recommendation regarding smoking for patients who suffered from a myocardial infarction?

A. smoking is allowed only with a morning coffee
B. immediate smoking cessation, if possible already during the hospitalisation

C. limiting smoking to 0,5 a pack a day

D. smoking is not associated with myocardial infarction, therefore there is no need for lifestyle modification in this area

1. Does reduction in body mass weight (in case of overweigh/ obesity) play a role in preventing the development of cardiovascular diseases?
   A. Yes
   B. No
2. Which of the following is the so called „bad cholesterol”, which can bild up in the vascular wall, forming atherosclerotic plaques?
   A. HDL
   B. LDL
   C. VLDL
3. Please mark the false statement regarding the role of physical activity in limiting the cardiovascular risk:
   A. Physical activity improves the lipid profile and decreases the concentration of LDL-C (“bad cholesterol”)

B. After suffering from myocardial infarction it is recommended to rest and resign from any physical activity for at least a month.

C. It is recommended to spend around 150 min/ per week (about 30 min, 3-5 times a week) on moderate physical activity (e.g. quick march, household chores )

D. The Patient’s physical activity should begin during hospitalisation, under specialist supervision.
E. If the patients experiences chest pai nor inadequate fatigue during training, the exercise should be ended and the patient should follow the doctors instructions.

1. Diet, which limits the cardiovascular risk is reach in:

A. polyunsaturated fatty acids– omega-3 and -6
B. pork, as a source of saturated fatty acids
C. salt
D. fruit juices, with high fructose concentrations

1. Which of the following are the non-traditional cardiovascular risk factors?
   A. obstructive sleep apnoea (OSA)

B. exposure to noise and air pollution
C. hormonal contraception
D. alcohol abuse
E. all answers are correct

Results:

Question 1: answer A - 1 point, answer B - 0 points

Question 2: no correct answer - 0 points

1-2 correct answers - 1 points

3-4 correct answers - 2 points

5-6 correct answers - 3 points

> 6 correct answers - 4 points

Question 3: answer C - 1 point, other answers - 0 points

Question 4: answer D - 1 point, other answers - 0 points

Question 5: answer B - 1 point, other answers - 0 points

Question 6: answer A - 1 point, answer B - 0 points

Question 7: Answer B - 1 point, other answers - 0 points

Question 8: Answer B - 1 point, other answers - 0 points

Question 9: answer A - 1 point, other answers - 0 points

Question 10: answer E - 1 point, other answers - 0 points
